# Studies with *β*-Oxoalkanonitriles: Simple Novel Synthesis of 3-[2,6-Diaryl-4- pyridyl]-3-oxopropanenitriles

**DOI:** 10.3390/molecules13123140

**Published:** 2008-12-15

**Authors:** Sayed M. Riyadh, Hamad M. Al-Matar, Mohamed H. Elnagdi

**Affiliations:** 1 Department of Chemistry, Faculty of Science, Cairo University, Giza-12613- Egypt. E-mail: riyadh1993@hotmail.com (S-M. R.); 2 Department of Chemistry, Faculty of Science, Kuwait University, Safat 13060 Kuwait, P.O. Box 5969. E-mail: shelmy1941@yahoo.com (M-H. E.)

**Keywords:** *β*-Oxoalkanonitriles, 3-Pyrazolylamine, 3-Isoxazolylamine, Phenacyl bromide, Ethyl cyanoacetate.

## Abstract

Heteroaromatization of ethyl 2-cyano-4-oxo-2-(2-oxo-2-arylethyl)-4-aryl-butanoates **3a,b** with ammonium acetate gave ethyl 2,6-diarylisonicotinates **4a,b**.Treatment of the latter with acetonitrile afforded novel *β*-oxoalkanonitriles **6a,b**. Reactions of **6a,b** with phenyl hydrazine and hydroxylamine gave the corresponding pyridyl aminopyrazoles **8a,b** and pyridyl aminoisoxazoles **10a,b**, respectively.

## Introduction

*β*-Oxoalkanonitriles are versatile reagents and their chemistry has received in the past [1] and continues to receive considerable attention [2,3,4,5,6,7,8,9]. In conjunction with our interest in utilizing oxoalkanonitriles to prepare azolylazines [10,11,12,13] a route to 3-(4-pyridyl)-3-oxopropanenitrile was needed. 4-Pyridyl derivatives possess many pharmacological activities [14], and they can also be used as *N*-donor ligands in complexation with metal ions with superior cytotoxicity towards bacteria [15]. 

## Results and Discussion

**Scheme 1:**
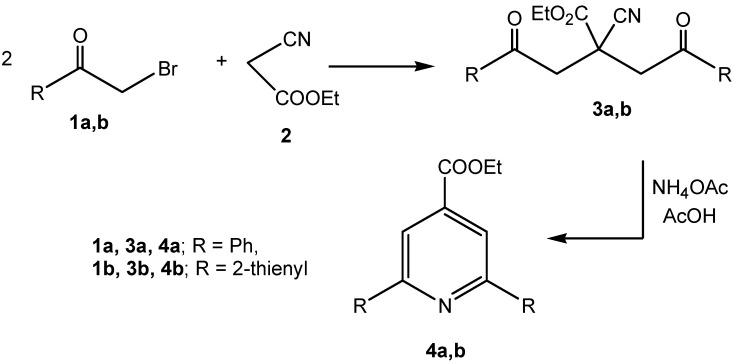
Synthetic pathway for preparation of compounds **4a,b**.

*β*-Oxoalkanonitriles are generally prepared *via*: i) acylation of active nitriles in the presence of suitable basic catalysts [16,17,18]; ii) reacting *α*-haloketones with cyanide ion [19], and iii) hydrolysis of *β*-enaminonitriles [7]. We decided to develop our synthesis *via* reaction of acetonitrile with ethyl 2,6-diarylisonicotinate. Although the parent ethyl 2,6-diphenylisonicotinate (**3a**) is a known compound, [20] the 2,6-diarylsubsubstituted derivatives have not, to our knowledge, been previously reported. Consequently, a method for their synthesis was developed. Reaction of phenacyl bromide (**1a**) with ethyl cyanoacetate (**2**) afforded the dialkylated derivative **3a** [21]. Similarly, **3b** was obtained by reacting **1b** with ethyl cyanoacetate. Refluxing **3a,b** in acetic acid in the presence of ammonium acetate afforded the target pyridines **4a,b** (Scheme 1).

**Scheme 2:**
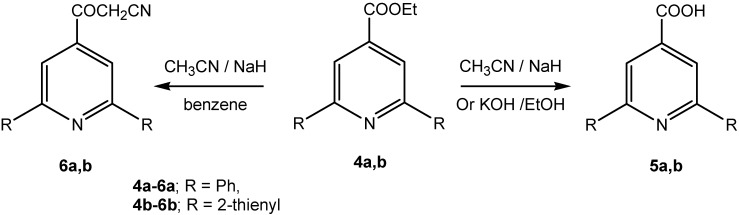
Synthetic pathway for preparation of compounds **5a,b** and **6a,b**.

Attempts to condense acetonitrile in an aqueous protic solvent with pyridines **4a,b** under different conditions afforded only the carboxylic acid derivatives **5a,b** [22]. However, the target *β*-oxo-alkanonitriles **6a,b** were obtained by reacting **4a,b** with acetonitrile in dry benzene in the presence of sodium hydride (Scheme 2). 

**Scheme 3:**
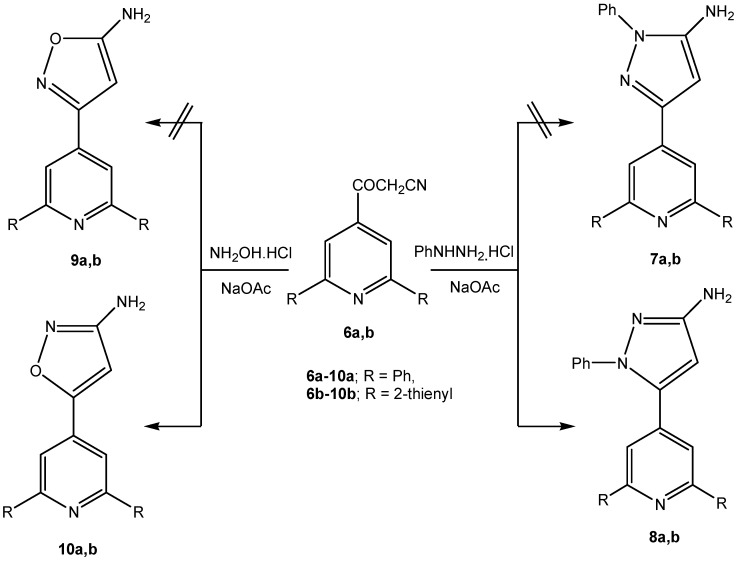
Reactions of *β*-oxoalkanonitriles **6a,b** with nitrogen nucleophiles.

As would be expected, *β*-oxoalkanonitriles **6a,b** reacted with phenyl hydrazine hydrochloride to yield the corresponding 5-aminopyrazoles **7a,b** or 3-aminopyrazoles **8a,b**. Despite literature reports [23,24], the *δ* value for the pyrazole ring H-4 for both isomers vary over the range between 5.3-6.1 ppm. To substantiate the regioselectivity of the reaction products, NOE difference experiments were performed, which showed that irradiation of the amino protons at *δ* 4.45 ppm did not enhance the aryl protons at *δ* 7.5 ppm and *vice versa*, irradiation of the *o*-aryl protons at *δ* 7.5 ppm did not enhance the amino protons. These results allowed us to conclude that the amino and aryl protons are not proximal (Scheme 3); that is, the compounds have the structures **8a,b**. Moreover, the reaction of *β*-oxoalkano-nitriles **6a,b** with hydroxylamine hydrochloride in the presence of sodium acetate could yield 5-aminoisoxazoles **9a,b** or the isomeric 3-aminoisoxazole structures **10a,b**. ^1^H-NMR revealed a singlet signal at *δ* = 7.00 ppm correlated to the isoxazole ring H-4. It was reported that the H-4 of 3-amino-isoxazole appears at lower field (*δ* ~ 6.1 ppm) than that of 5-aminoisoxazole (*δ* ~ 5.5 ppm) [25,26]. Moreover, ^15^N, 1H-heteronuclear multiple bond correlation (HMBC) of the product indicated that amino proton at *δ* 5.85 ppm has a cross peak at *δ* 350 ppm (^3^*J* coupling). These results indicated that structures **10a,b** are the most probable for the reaction products (cf. Scheme 3). The reaction of 2-substituted-3-oxoalkanonitriles with hydroxylamine hydrochloride in presence of sodium acetate has been reported by Elnagdi *et al*. [27] to yield amidoximes that cyclised into 3-aminoisoxazoles. On the other hand formation of 5-aminoisoxazoles from the reaction of isonicotinylacetonitriles with hydroxylamine hydrochloride was reported in the patent literature [28,29], although there is no mention of added base in those cases.

## Conclusions

A novel route for the synthesis of *β*-oxoalkanonitriles has been developed. The products from this synthesis were further reacted with nitrogen nucleophiles to give azoles. The features of the present method include the ready availability of the starting materials, mild reaction conditions, and the simplicity of the workup. 

## Experimental

### General

Melting points were recorded on a Gallenkamp apparatus and are uncorrected. Infrared spectra (KBr) were determined on a Perkin-Elmer 2000 FT-IR system. ^1^H-NMR spectra were recorded on a Bruker DPX 600 MHz superconducting spectrometer using DMSO-d_6_ as solvent and TMS as internal standard. Mass spectra were measured on MS 30 and MS 9 (AEI) spectrometers, operating at EI 70 ev. Elemental analyses were measured by means of LECO CHNS-932 Elemental Analyzer. 

### Synthesis of ethyl 2-cyano-4-oxo-2-(2-oxo-2-arylethyl)-4-arylbutanoates **3a,b**

To a stirred solution of *α*-haloketone (20 mmol) in ethanol (50 mL) containing ethyl cyanoacetate (10 mmol) was added potassium hydroxide solution (10 mmol, 0.56 g, dissolved in 20 mL H_2_O). The mixture was stirred for 30 minutes and acidified. The solid precipitate was collected by filtration and recrystallized from hexane/EtOAc (3:1).

*Ethyl 2-cyano-4-oxo-2-(2-oxo-2-phenylethyl)-4-phenylbutanoate* (**3a**): Yield: 2.79 g (80%); mp 140 ^o^C (lit. [21] mp 141 ^o^C); IR υ = 2250, (CN), 1722 (CO, ester), 1689 (CO, benzoyl) cm^-1^; ^1^H-NMR *δ* = 1.22 (t, 3H, CH_3_), 3.95 (d, 2H, CH_2_), 4.03 (d, 2H, CH_2_), 4.19 (q, 2H, CH_2_), 7.56-8.01 (m, 10H, Ar-H) ppm; MS, *m/z* (%) 349 (M^+^, 20), 276 (30), 244 (50), 105 (100), 77 (80); Anal. Calcd. for C_21_H_19_NO_4_: C, 72.19; H, 5.48; N, 4.01. Found: C, 72.44; H, 5.24; N, 4.05.

*Ethyl 2-cyano-4-oxo-2-[(2-oxo-2-(2-thienyl)ethyl]-4-(2-thienyl)butanoate* (**3b**): Yield: 2.7 g (75%); mp 102 ^o^C; IR υ = 2251, (CN), 1730 (CO, ester), 1668 (CO, thienoyl) cm^-1^; ^1^H-NMR *δ* = 1.21 (t, 3H, CH_3_), 3.84 (d, 2H, CH_2_), 3.96 (d, 2H, CH_2_), 4.17 (q, 2H, CH_2_), 7.28-8.10 (m, 6H, Ar-H) ppm; MS, *m/z* (%) 361 (M^+^, 55), 288 (70), 250 (60), 126 (80), 111 (100), 83 (30); Anal. Calcd. for C_17_H_15_NO_4_S_2_: C, 56.49; H, 4.18; N, 3.88; S, 17.74. Found: C, 56.40; H, 4.11; N, 3.95; S, 18.01.

### Synthesis of ethyl 2,6-diarylisonicotinates **4a,b**

A mixture of ethyl 2-cyano-4-oxo-2-(2-oxo-2-arylethyl)-4-arylbutanoate (10 mmol) and ammonium acetate (15 mmol) in glacial acetic acid (20 mL) was refluxed for 8 hours and poured into water. The solid precipitate was collected by filtration and purified by long column chromatography [eluent: hexane/EtOAc (3:1)].

*Ethyl 2,6-diphenylisonicotinate* (**4a**): Yield: 1.82 g (60%); mp 98 ^o^C (lit. [20] mp 99 ^o^C); IR υ = 1716 (CO, ester), 1561 (C=N), 1250 (C-O) cm^-1^; ^1^H-NMR *δ* = 1.39 (t, 3H, CH_3_), 4.44 (q, 2H, CH_2_), 7.52-8.27 (m, 12H, Ar-H) ppm; MS, *m/z* (%) 303 (M^+^, 90), 231 (100), 127 (50), 77 (20); Anal. Calcd. for C_20_H_17_NO_2_: C, 79.19; H, 5.65; N, 4.62. Found: C, 78.96; H, 5.90; N, 4.66.

*Ethyl 2,6-di(2-thienyl)isonicotinate* (**4b**): Yield: 1.89 g (60%); mp 113 ^o^C; IR υ = 1714 (CO, ester), 1565 (C=N), 1250 (C-O) cm^-1^; ^1^H-NMR *δ* = 1.39 (t, 3H, CH_3_), 4.42 (q, 2H, CH_2_), 7.20-8.10 (m, 8H, Ar-H) ppm; MS, *m/z* (%) 315 (M^+^, 100), 243 (20), 133 (25), 89 (15); Anal. Calcd. for C_16_H_13_NO_2_S_2_: C, 60.93; H, 4.15; N, 4.44; S, 20.33. Found: C, 60.63; H, 4.45; N, 4.74; S, 20.58.

### Synthesis of 2,6-diarylisonicotinic acids **5a,b**

A solution of ethyl 2,6-diarylisonicotinate (10 mmol) in ethanol (20 mL) was treated with potassium hydroxide solution (15 mmol in 10 mL water) and refluxed for 4 hours. The reaction mixture was poured into ice/HCl. The solid precipitate was collected by filtration and recrystallized from ethanol.

*2,6-Diphenylisonicotinic acid* (**5a**): Yield: 1.92 g (70%); mp 265 ^o^C (lit. [22] mp 263 ^o^C); IR υ = 3500-2600 (br, OH acid), 1698 (CO, acid), 1555 (C=N), 1279 (C-O) cm^-1^; ^1^H-NMR *δ* = 7.49-8.26 (m, 12H, Ar-H), 13.98 (COOH) ppm; MS, *m/z* (%) 275 (M^+^, 100), 231 (40), 127 (10), 77 (10); Anal. Calcd. for C_18_H_13_NO_2_: C, 78.53; H, 4.76; N, 5.09. Found: C, 78.38; H, 4.68; N, 5.01.

*2,6-Di(2-thienyl)isonicotinic acid* (**5b**): Yield: 1.72 g (60%); mp 251 ^o^C; IR υ = 3500-2500 (br, OH acid), 1700 (CO, acid), 1561 (C=N), 1271 (C-O) cm^-1^; ^1^H-NMR *δ* = 7.31-8.21 (m, 8H, Ar-H), 13.86 (COOH) ppm; MS, *m/z* (%) 287 (M^+^, 100), 242 (40), 83 (10); Anal. Calcd. for C_14_H_9_NO_2_S_2_: C, 58.52; H, 3.16; N, 4.87; S, 22.32. Found: C, 58.33; H, 3.11; N, 4.64; S, 22.51.

### Synthesis of β-oxoalkanonitriles **6a,b**

A mixture of ethyl 2,6-diarylisonicotinate (1 mmol), dry acetonitrile (2 mmol), and sodium hydride (20 mmol) in dry benzene (20 mL) was refluxed for 4 hours and poured into water, extracted by ethyl acetate. The solvent was evaporated under vacuum and the residue was purified by long column chromatography [eluent: hexane/EtOAc (3:1)].

*3-(2,6-Diphenyl-4-pyridyl)-3-oxopropanenitrile* (**6a**): Yield: 0.149 g (50%); mp 180 ^o^C; IR υ = 2217 (CN), 1710 (CO), 1594 (C=N) cm^-1^; ^1^H-NMR *δ* = 4.98 (s, 2H, CH_2_), 7.36-8.30 (m, 12H, Ar-H) ppm; MS, *m/z* (%) 298 (M^+^, 100), 270 (15), 127 (65), 77 (70); Anal. Calcd. for C_20_H_14_N_2_O: C, 80.52; H, 4.73; N, 9.39. Found: C, 80.38; H, 4.61; N, 9.08.

*3-[2,6-Di(2-thienyl)-4-pyridyl]-3-oxopropanenitrile* (**6b**): This compound was obtained in 0.155 g (50%), mp 192 ^o^C; IR (KBr) υ = 2214 (CN), 1703 (CO), 1592 (C=N) cm^-1^; ^1^H NMR (DMSO-d_6_) *δ* = 4.41 (s, 2H, CH_2_), 7.02-8.10 (m, 8H, Ar-H) ppm; MS, *m/z* (%) 310 (M^+^, 100), 282 (35), 139 (20), 83 (15). *Anal*. Calcd. for C_16_H_10_N_2_OS_2_: C, 61.91; H, 3.25; N, 9.03; S, 20.66. Found: C, 61.86; H, 3.14; N, 9.11; S, 20.51.

### Reactions of β-oxoalkanonitriles with nitrogen nucleophiles

A mixture of *β*-oxoalkanonitriles (1 mmol) in dioxane (20 mL) in presence of anhydrous sodium acetate (2 mmol) and hydroxylamine hydrochloride or phenylhydrazine hydrochloride (1 mmol) was refluxed for 4 hours. After pouring into water, the solid precipitate was collected by filtration and recrystallized from hexane/ethyl acetate mixture (3:1). 

*5-(2,6-Diphenyl-4-pyridyl)-1-phenyl-1H-3-pyrazolylamine* (**8a**): Yield: 0.194 g (50%); mp 210 ^o^C; IR υ = 3424, 3280 (NH_2_), 1602 (C=N) cm^-1^; ^1^H-NMR *δ* = 4.45 (s, 2H, NH_2_), 6.93 (s, 1H, pyrazole-H), 7.34-8.30 (m, 17H, Ar-H) ppm; ^13^C-NMR *δ* = 97.3, 117.8, 118.1, 126.3, 127.1, 127.9, 128.3, 129.1, 130.2, 138.6, 139.3, 144.3, 153.7, 161.5 (Ar-Cs); MS, *m/z* (%) 388 (M^+^, 100), 284 (5), 230 (10), 77 (15); Anal. Calcd. for C_26_H_20_N_4_: C, 80.39; H, 5.19; N, 14.42. Found: C, 80.17; H, 5.49; N, 14.51.

*5-[2,6-Di(2-thienyl)-4-pyridyl)-1-phenyl-1H-3-pyrazolylamine* (**8b**): Yield: 0.20 g (50%); mp 235 ^o^C; IR υ = 3410, 3271 (NH_2_), 1601 (C=N) cm^-1^; ^1^H-NMR *δ* = 4.43 (s, 2H, NH_2_), 6.93 (s, 1H, pyrazole-H), 7.20-8.30 (m, 13H, Ar-H) ppm; ^13^C-NMR *δ* = 96.8, 117.1, 118.4, 125.7, 126.3, 126.9, 127.1, 128.3, 129.4, 130.2, 134.1, 144.5, 152.7, 161.1 (Ar-Cs); MS, *m/z* (%) 400 (M+, 40), 296 (100), 241 (40), 77 (15); Anal. Calcd. for C_22_H_16_N_4_S_2_: C, 65.97; H, 4.03; N, 13.99; S, 16.01. Found: C, 66.13; H, 4.15; N, 13.81; S, 15.91.

*5-(2,6-Diphenyl-4-pyridyl)-3-isoxazolylamine* (**10a**): Yield: 0.188 g (60%); mp 179 ^o^C; IR *υ* = 3349, 3297 (NH_2_), 1640 (C=N) cm^-1^; ^1^H-NMR *δ* = 5.85 (s, 2H, NH_2_), 7.00 (s, 1H, isoxazole-H), 7.48-8.29 (m, 12H, Ar-H) ppm; ^13^C-NMR *δ* = 98.3, 124.2, 126.1, 127.3, 128.4, 136.5, 142.6, 148.3, 162.8, 167.2 (Ar-Cs); MS, *m/z* (%) 314 (M++1, 35), 313 (M+, 30), 275 (100), 127 (25), 77 (15); Anal. Calcd. for C_20_H_15_N_3_O: C, 76.66; H, 4.82; N, 13.41. Found: C, 76.82; H, 5.05; N, 13.68.

*5-[2,6-Di(2-thienyl)-4-pyridyl]-3-isoxazolylamine* (**10b**): Yield: 0.195 g (60%); mp 192 ^o^C; IR υ = 3342, 3291 (NH_2_), 1645 (C=N) cm^-1^; ^1^H-NMR *δ* = 5.85 (s, 2H, NH_2_), 7.21 (s, 1H, isoxazole-H), 7.22-8.10 (m, 8H, Ar-H) ppm; ^13^C-NMR *δ* = 98.8, 125.4, 126.8, 127.5, 128.4, 138.5, 142.2, 149.1, 163.8, 168.1 (Ar-Cs); MS, *m/z* (%) 325 (M^+^, 100), 287 (35), 133 (25), 83 (15); Anal. Calcd. for C_16_H_11_N_3_OS_2_: C, 59.06; H, 3.41; N, 12.91; S, 19.71. Found: C, 59.13; H, 3.34; N, 12.81; S, 19.51.
